# Compare the efficacy of antifungal agents as primary therapy for invasive aspergillosis: a network meta-analysis

**DOI:** 10.1186/s12879-024-09477-9

**Published:** 2024-06-12

**Authors:** Ao Liu, Liubo Xiong, Lian Wang, Han Zhuang, Xiao Gan, Mengying Zou, Xiaoming Wang

**Affiliations:** Department of Respiratory Medicine, Chengdu BOE hospital, Chengdu, Sichuan Province 610000 China

**Keywords:** Invasive aspergillosis, Antifungal agents, Primary therapy, Network meta-analysis

## Abstract

**Background:**

Several antifungal agents are available for primary therapy in patients with invasive aspergillosis (IA). Although a few studies have compared the effectiveness of different antifungal agents in treating IA, there has yet to be a definitive agreement on the best choice. Herein, we perform a network meta-analysis comparing the efficacy of different antifungal agents in IA.

**Methods:**

We searched PubMed, Embase, and the Cochrane Central Register of Controlled Clinical Trials databases to find studies (both randomized controlled trials [RCTs] and observational) that reported on treatment outcomes with antifungal agents for patients with IA. The study quality was assessed using the revised tool for risk of bias and the Newcastle Ottawa scale, respectively. We performed a network meta-analysis (NMA) to summarize the evidence on antifungal agents’ efficacy (favourable response and mortality).

**Results:**

We found 12 studies (2428 patients) investigating 11 antifungal agents in the primary therapy of IA. There were 5 RCTs and 7 observational studies. When treated with monotherapy, isavuconazole was associated with the best probability of favourable response (SUCRA, 77.9%; mean rank, 3.2) and the best reduction mortality against IA (SUCRA, 69.1%; mean rank, 4.1), followed by voriconazole and posaconazole. When treated with combination therapy, Liposomal amphotericin B plus caspofungin was the therapy associated with the best probability of favourable response (SUCRA, 84.1%; mean rank, 2.6) and the best reduction mortality (SUCRA, 88.2%; mean rank, 2.2) against IA.

**Conclusion:**

These findings suggest that isavuconazole, voriconazole, and posaconazole may be the best antifungal agents as the primary therapy for IA. Liposomal amphotericin B plus caspofungin could be an alternative option.

**Supplementary Information:**

The online version contains supplementary material available at 10.1186/s12879-024-09477-9.

## Introduction

Invasive aspergillosis (IA) is a severe infection with high morbidity and mortality, primarily affecting immunocompromised individuals, including transplant recipients, advanced HIV patients, and those with primary immunodeficiency or hematologic malignancies [1, 2]. Recent studies suggest a susceptibility to IA in individuals with severe viral respiratory infections like influenza and COVID-19 [3, 4]. With over 200,000 cases annually worldwide [5], IA previously had an 80% mortality rate in immunocompromised individuals [6], but with triazoles as primary therapy, this has decreased to less than 30% [7].

Voriconazole and isavuconazole are the top recommended antifungal agents for primary therapy according to international guidelines [8, 9], with posaconazole showing non-inferiority to voriconazole in a recent randomized clinical trial (RCT) for all-cause mortality [10]. Echinocandins, though static against aspergillus, are considered for primary and salvage therapy in combination with other antifungals due to low toxicity [11–15], but RCTs evaluating monotherapy with echinocandins are lacking. Lipid formulations of amphotericin B are less toxic than amphotericin B deoxycholate but show less efficacy than voriconazole [16]. Azole resistance among aspergillus is a growing concern, with prevalence ranging from 3–30% [17, 18]. Considering these challenges and the high mortality of IA, combination therapies like liposomal amphotericin B plus echinocandins may offer enhanced survival rates as primary therapy [13, 15]. 

Thus, the best choice of antifungal agents for primary therapy in patients with IA remains unclear. Network meta-analysis (NMA) provides the relative effectiveness of various interventions. We conducted a NMA to evaluate the relative efficacy of different treatments in IA.

## Methods

### Protocol and registration

This NMA was conducted in accordance with the Preferred Reporting Items for Systematic Reviews and Meta-analyses for Network Meta-analysis (PRISMA-NMA) reporting guideline [19]. This protocol has been registered at PROSPERO under registration number CRD42023436758.

### Literature search

We searched the Pubmed, EMBASE, and the Cochrane Central Register of Controlled Clinical Trials to collect all published evidence from RCTs and observational studies from inception to 01 July 2023 that assessed primary therapy in patients with IA. The detailed search strategy is presented in the supplemental eTable 1 in the Supplement. The reference lists from all included studies were also screened to identify potentially relevant evidence.

### Study inclusion and exclusion criteria

The inclusion criteria were (a) RCTs comparing different antifungal agents or placebo in patients with IA; (b) RCTs with reported available data that measured response rate and/or mortality rate; (c) observational studies providing the outcomes with at least two different antifungal agents; (d) published in only English language. The exclusion criteria were (a) abstracts, comments, editorials, case reports, and reviews; (b) studies using a single antifungal agent without a control arm or with different dose; (c) studies including patients with allergic bronchopulmonary aspergillosis or chronic pulmonary aspergillosis.

### Data extraction

From each relevant study, two authors (L.-B.X. and L.W.) independently extracted the following data: authors’ names, year of publication, number of patients, age, use, and dosage of drugs. Extracted outcomes included favourable therapy response and mortality of different antifungal agents used. Any differences in the data extraction process were resolved by discussion.

### Quality assessment

Two authors (H.Z. and X.G.) independently participated in the quality assessment, and disagreements were resolved by discussion. The quality of the evidence was assessed using the revised tool for risk of bias (ROB2) and the Newcastle-Ottawa scale (NOS)in RCTs and observational studies, respectively [20, 21]. 

### Outcome measure

The outcomes including favourable response and mortality were to compare the efficacy of antifungal agents in treating IA. Favourable response was defined based on the criteria used by the authors, which included complete response, partial response, or stable disease.

### Statistical analysis

We compared different antifungal agents through network meta-analyses performed under a frequentist framework using a random-effects model. The analysis utilized the network and mvmeta packages in Stata (StateCorp, USA) [22]. We assessed the results of each study using the relative risk (RR) with 95% confidence intervals (CIs). If the 95% CI of an RR did not include 1, it indicated a significant association. We utilized forest plots and league tables to present the network meta-analysis findings. Additionally, we ranked each agent individually for each outcome using the surface under the cumulative ranking curve (SUCRA) [23]. A higher SUCRA value indicates a better rank. To ensure reliability and validity, we addressed inconsistencies and heterogeneity in the comparative studies of various treatments when assessing the networks [24]. The overall and loop inconsistencies were evaluated [22, 25]. We used the restricted maximum likelihood method to assess heterogeneity. A τ2 value less than 0.1 indicated a very low level of heterogeneity, and a τ2 value from 0.1 to 0.5 indicated a reasonable level; a τ2 value greater than 0.5 indicated a high heterogeneity [26]. We also used a funnel plot to describe small-study effects and tested each plot with the Egger’s test. A 2-sided P-value less than 0.05 is statistically significant.

## Results

### Characteristics of the studies

**Fig. 1:**
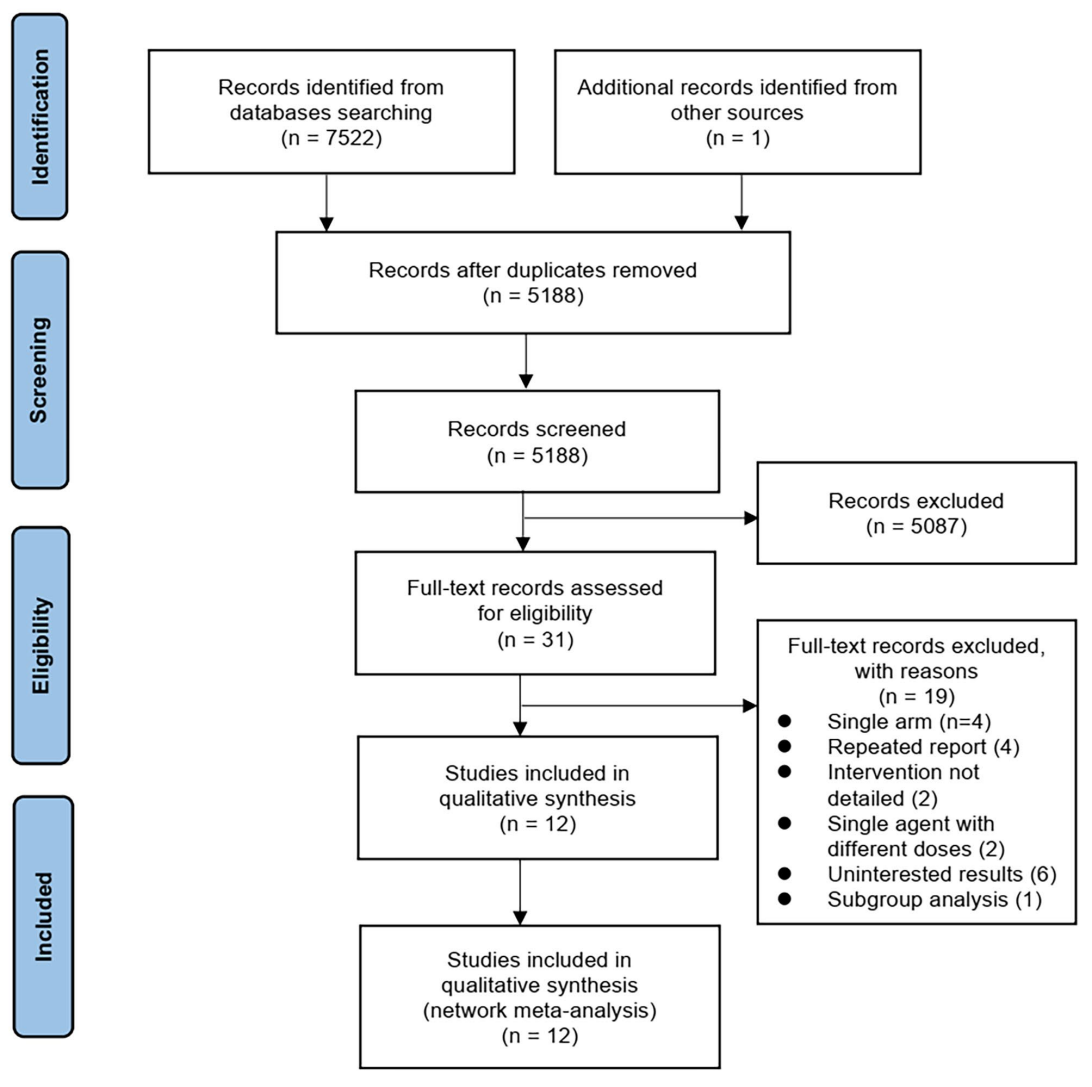
PRISMA Flowchart

The flowchart of study selection for this NMA is shown in Fig. 1. In total, 5 RCTs and 7 observational studies with 2428 patients were included [10, 13–15, 27–34], including 11 groups: amphotericin B colloidal dispersion, amphotericin B deoxycholate, liposomal amphotericin B, amphotericin B lipid complex, voriconazole, posaconazole, caspofungin, isavuconazole, liposomal amphotericin B plus caspofungin, voriconazole plus caspofungin, voriconazole plus anidulafungin. The basic characteristics of the included studies are summarized in Table 1. The quality of included studies is shown in supplemental eTable 2 and eFigure 2. ROB2 scored 1, 2, and 3, respectively, mean low risk, some concerns, and high risk.

**Table 1:** Basic Characteristics of Included Studies

Randomized clinical trial											
Author/year	**Study design**	**Country**	**Population**	**Definition of favourable response**	**Interventions**			**Patients, No.**	**Mean age,** **(range), y**	**Follow-up**	**ROB2**
					**A**	**B**	**C**				
Bowden, et al. [29] 2002	RCT	International	Patients with immune-compromising conditions	Complete response: normalization of clinical features and radiology. Partial response: improvement of clinical features and radiology.	ABCD 6 mg/kg IV QD	AmB 1-1.5 mg/kg IV QD		174	46 (0–81)	NR	2
Herbrecht, et al. [32] 2015	RCT	International	Patients with immune-compromising conditions	Complete response: normalization of clinical features and ≥ 90% improvement in radiology. Partial response: improvement of clinical features and ≥ 50% improvement in radiology.	VOCZ 6 mg/kg IV BID d1, then 4 mg/kg IV BID d2-8, then 200 mg PO BID	AmB 1-1.5 mg/kg IV QD		237	49 (12–79)	12 weeks	1
Marr, et al. [13] 2015	RCT	International	Patients with hematologic malignancies and hematopoietic cell transplantation	Complete response: normalization of clinical features and ≥ 90% improvement in radiology. Partial response: improvement of clinical features and ≥ 50% improvement in radiology.	VOCZ 6 mg/kg IV BID d1, then 4 mg/kg IV BID d2-8, then 300 mg PO bid + ANFG 200 mg IV QD d1, then 100 mg IV QD.	VOCZ 6 mg/kg IV BID d1, then 4 mg/kg IV BID d2-8, then 300 mg PO BID		277	52 (18–83)	12 weeks	1
Maertens, et al. [33] 2016	RCT	International	Patients with immune-compromising conditions	Favourable response: improvement of clinical features and ≥ 50% improvement in radiology.	ISCZ 200 mg IV TID d1-2, then 200 mg IV/PO QD	VOCZ 6 mg/kg IV BID d1, then 4 mg/kg IV BID d2, then 200 mg IV/PO BID		272	NR	12 weeks	1
Maertens, et al. [10] 2021	RCT	International	Patients with immune-compromising conditions	Complete response: normalization of clinical features and radiology. Partial response: improvement of clinical features and radiology.	POCZ 300 mg IV/PO BID d1, then 300 mg IV or PO QD	VOCZ 6 mg/kg IV or 300 mg PO BID d1, then 4 mg/kg IV or 200 mg PO BID		334	53	12 weeks	1
Observational study											
Author/year	**Study design**	**Country**	**Population**	**Definition of favourable response**	**Interventions**			**Patients, No.**	**Mean age,** **(range), y**	**Follow-up**	**NOS**
					**A**	**B**	**C**				
White, et al. [27] 1997	Retrospective	United States	​Patients with hematological or organ malignancies	Complete response: normalization of clinical features and radiology. Partial response: improvement of clinical features + stabilization or improvement in radiology.	ABCD 0.5-8 mg/kg IV QD	AmB 0.1–1.4 mg/kg IV QD		343	41 (0–79)	NR	7
Leenders, et al. [28] 1998	Prospective	International	Neutropenic patients	Complete response: normalization of clinical features + improvement in radiology. Partial response: improvement of clinical features + stabilization or improvement in radiology.	L-AmB 5 mg/kg IV QD	AmB 1 mg/kg IV QD		55	50 (18–74)	NR	9
Singh, et al. [15] 2006	Prospective	International	Solid organ transplant recipients	Complete response: normalization of clinical features and ≥ 90% improvement in radiology. Partial response: improvement of clinical features and ≥ 50% improvement in radiology.	VOCZ 6 mg/kg IV Q12H d1, then 4 mg/kg Q12H + CAFG 70 mg IV QD d1, then 50 mg IV QD	L-AmB 5-7.4 mg/kg IV QD		87	50 (19–68)	90 days	9
Caillot, et al. [14] 2007	Prospective	France	patients with hematologic malignancies	Complete response: normalization of clinical features and near normalization of radiology. Partial response: meaningful improvement of clinical features and ≥ 50% improvement in radiology.	L-AmB 3 mg/kg IV QD + CAFG 70 mg IV QD d1, then 50 mg IV QD	L-AmB 10 mg/kg IV QD		30	54 (16–75)	12 weeks	9
Hachem, et al. [30] 2008	Retrospective	United States	Patients with hematologic malignancy	Favourable response: improvement of clinical features and ≥ 50% improvement in radiology.	ABLC 5–10 mg/kg IV QD	L-AmB 5–10 mg/kg IV QD		122	NR	NR	8
Pagano, et al. [31] 2010	Prospective		Patients with acute myeloid leukemia	Complete response: normalization of clinical features and ≥ 90% improvement in radiology. Partial response: improvement of clinical features and ≥ 50% improvement in radiology.	L-AmB	Voriconazole	Caspofungin	103	NR	120 days	9
Cheng, et al. [34] 2020	Retrospective	United States	Patients with immune-compromising conditions	Complete response: normalization of clinical features and radiology. Partial response: improvement of clinical features and radiology.	Voriconazole	Isavuconazole		40	62 (20–80)	12 weeks	8

Abbreviations: AmB, amphotericin B deoxycholate; L-AmB, liposomal amphotericin B; ABCD, amphotericin B colloidal dispersion; ABLC, amphotericin B lipid complex; VOCZ, voriconazole; POCZ, posaconazole; CAFG, caspofungin; ISCZ, isavuconazole; ANFG, anidulafungin; ROB2, revised tool for risk of bias; NOS, Newcastle-Ottawa scale; D, day; QD, every day; BID, twice a day; TID, three times a day; Q12H, every 12 h; IV, intravenous; PO, oral; NR, not reported; RCT, randomized clinical trial

### Network geometry and synthesis of results

**Fig. 2:**
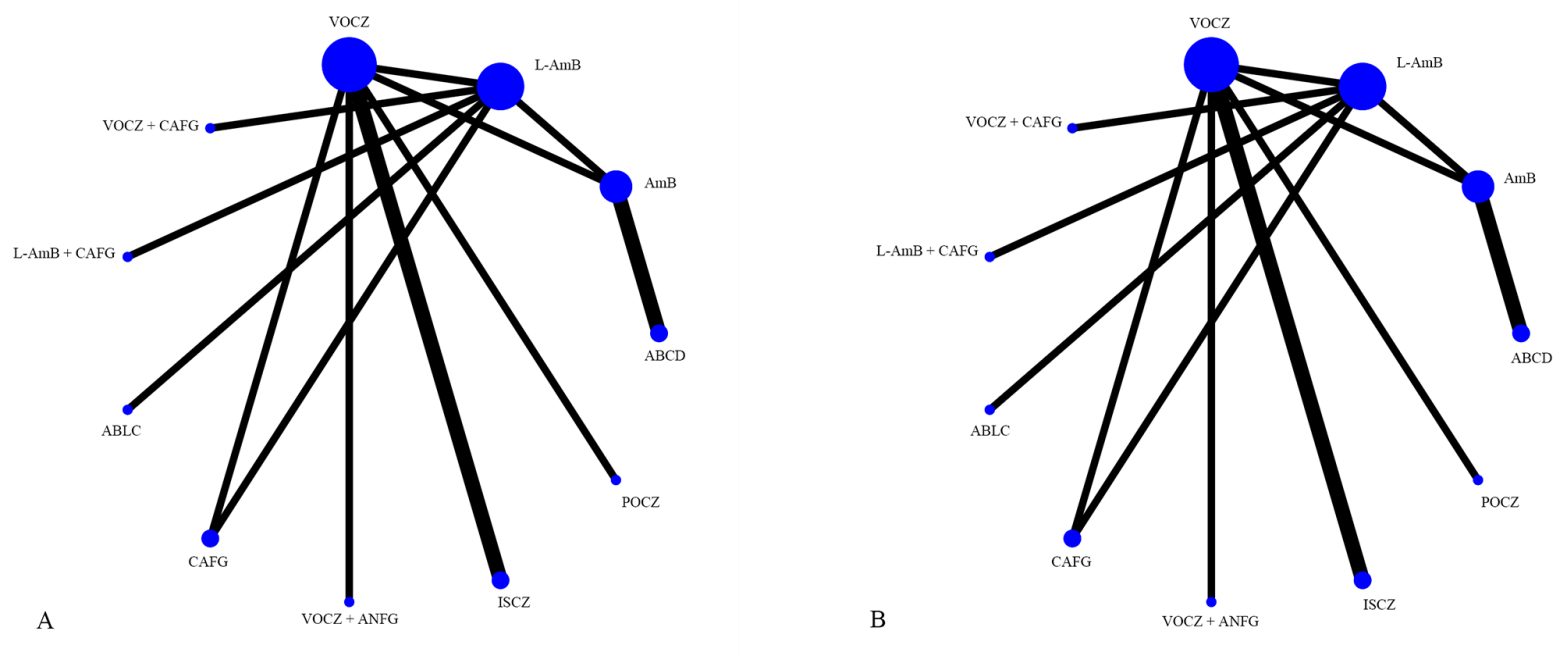
Network Geometry of All Outcomes. Network plot for response (panel **A**) and mortality (panel **B**) of antifungal agents for primary therapy of invasive aspergillosis. When study for direct comparisons exist, this is shown by connections between nodes. The size of the node represents the number of studies with a particular drug. The thickness of lines connecting nodes represents the number of trials for that comparison. AmB, amphotericin B deoxycholate; L-AmB, liposomal amphotericin B; ABCD, amphotericin B colloidal dispersion; ABLC, amphotericin B lipid complex; VOCZ, voriconazole; POCZ, posaconazole; CAFG, caspofungin; ISCZ, isavuconazole; ANFG, anidulafungin

The network geometry for each outcome is shown in Fig. 2: favourable response rates included 11 groups, 12 studies, and 2043 patients; mortality rates included 11 groups, 12 studies, and 2074 patients. Indirect and mixed-treatment comparisons are shown as forest plots (eFigure 2 in the Supplement).

The SUCRA value and rank of each agent for each outcome are shown in Table 2. Regarding response rate, combination therapy of liposomal amphotericin B plus caspofungin was the approach with the highest ranking (SUCRA, 84.1%; mean rank, 2.6), followed by isavuconazole (SUCRA, 77.9%; mean rank, 3.2), voriconazole (SUCRA, 59.1%; mean rank, 5.1), voriconazole plus caspofungin (SUCRA, 58.7%; mean rank, 5.1), posaconazole (SUCRA, 56.5%; mean rank, 5.3). Amphotericin B deoxycholate ranked the lowest (SUCRA, 17.7%; mean rank, 9.2). Compared to voriconazole, liposomal amphotericin B plus caspofungin show a trend improving favourable response (RR, 1.84; 95% CI, 0.47–7.21), but the difference is not significant. Isavuconazole (RR, 1.41; 95% CI, 0.69–2.87), posaconazole (RR, 0.99; 95% CI, 0.45–2.21), voriconazole plus caspofungin (RR, 1.01; 95% CI, 0.34–2.95) didn’t show a significant difference compared with voriconazole (Table 3).

**Table 2:** SUCRA Values and Mean Rank for All Outcomes

Treatment	Response rate		Mortality rate	
	SUCRA, %	MeanRank	SUCRA, %	MeanRank
L-AmB + CAFG	84.1^a^	2.6	88.2^a^	2.2
ISCZ	77.9	3.2	69.1	4.1
VOCZ	59.2	5.1	57.7	5.2
VOCZ + CAFG	58.7	5.1	66.9	4.3
POCZ	56.5	5.3	48.4	6.2
ABCD	47.1	6.3	35.8	7.4
VOCZ + ANFG	42.4	6.8	79.1	3.1
ABLC	38.7	7.1	40	7
L-AmB	35.2	7.5	36.8	7.3
CAFG	32.4	7.8	12.9	9.7
AmB	17.7	9.2	15	9.5

Abbreviations: AmB, amphotericin B deoxycholate; L-AmB, liposomal amphotericin B;

ABCD, amphotericin B colloidal dispersion; ABLC, amphotericin B lipid complex;

VOCZ, voriconazole; POCZ, posaconazole; CAFG, caspofungin; ISCZ, isavuconazole;

ANFG, anidulafungin

a Ranking first among agents

**Table 3:** League Tables of All Outcomes

**Panel A.** Response										
L-AmB + CAFG	0.76 (0.16,3.57)	0.54 (0.14,2.13)	0.55 (0.13,2.35)	0.54 (0.11,2.63)	0.45 (0.10,2.10)	0.43 (0.09,2.14)	0.38 (0.06,2.38)	0.40 (0.12,1.31)	0.38 (0.09,1.59)	0.31 (0.08,1.22)
1.31 (0.28,6.10)	ISCZ	0.71 (0.35,1.45)	0.72 (0.20,2.60)	0.71 (0.24,2.06)	0.59 (0.18,1.95)	0.57 (0.19,1.69)	0.50 (0.09,2.72)	0.52 (0.20,1.39)	0.49 (0.17,1.44)	0.40 (0.15,1.08)
1.84 (0.47,7.21)	1.41 (0.69,2.87)	VOCZ	1.01 (0.34,2.95)	0.99 (0.45,2.21)	0.83 (0.32,2.19)	0.80 (0.35,1.82)	0.70 (0.15,3.28)	0.74 (0.38,1.44)	0.69 (0.31,1.55)	0.56 (0.28,1.12)
1.82 (0.43,7.82)	1.39 (0.38,5.06)	0.99 (0.34,2.90)	VOCZ + CAFG	0.99 (0.26,3.76)	0.83 (0.23,2.98)	0.79 (0.20,3.07)	0.70 (0.14,3.52)	0.73 (0.32,1.69)	0.69 (0.21,2.21)	0.56 (0.19,1.66)
1.85 (0.38,9.01)	1.41 (0.48,4.13)	1.01 (0.45,2.24)	1.01 (0.27,3.87)	POCZ	0.84 (0.24,2.94)	0.80 (0.25,2.53)	0.71 (0.13,4.01)	0.74 (0.26,2.10)	0.70 (0.22,2.16)	0.57 (0.20,1.63)
2.21 (0.48,10.24)	1.69 (0.51,5.56)	1.20 (0.46,3.16)	1.21 (0.34,4.36)	1.19 (0.34,4.19)	ABCD	0.96 (0.27,3.42)	0.85 (0.16,4.58)	0.88 (0.34,2.33)	0.83 (0.26,2.67)	0.68 (0.34,1.34)
2.30 (0.47,11.37)	1.76 (0.59,5.24)	1.25 (0.55,2.86)	1.26 (0.33,4.90)	1.25 (0.39,3.94)	1.04 (0.29,3.72)	VOCZ + ANFG	0.88 (0.15,5.06)	0.92 (0.32,2.67)	0.87 (0.27,2.74)	0.71 (0.24,2.07)
2.61 (0.42,16.17)	2.00 (0.37,10.85)	1.42 (0.31,6.60)	1.43 (0.28,7.21)	1.41 (0.25,7.98)	1.18 (0.22,6.40)	1.13 (0.20,6.48)	ABLC	1.04 (0.26,4.16)	0.98 (0.20,4.89)	0.80 (0.17,3.76)
2.50 (0.76,8.22)	1.91 (0.72,5.08)	1.36 (0.69,2.66)	1.37 (0.59,3.17)	1.35 (0.48,3.84)	1.13 (0.43,2.98)	1.08 (0.37,3.14)	0.96 (0.24,3.82)	L-AmB	0.94 (0.42,2.13)	0.77 (0.38,1.53)
2.66 (0.63,11.25)	2.03 (0.70,5.94)	1.45 (0.65,3.23)	1.46 (0.45,4.70)	1.44 (0.46,4.47)	1.20 (0.37,3.88)	1.15 (0.36,3.65)	1.02 (0.20,5.08)	1.06 (0.47,2.41)	CAFG	0.82 (0.31,2.11)
3.26 (0.82,12.92)	2.49 (0.93,6.70)	1.77 (0.89,3.52)	1.79 (0.60,5.30)	1.76 (0.61,5.06)	1.48 (0.75,2.92)	1.41 (0.48,4.14)	1.25 (0.27,5.87)	1.30 (0.65,2.61)	1.23 (0.47,3.18)	AmB
**Panel B.** Mortality										
L-AmB + CAFG	3.99 (0.19,83.83)	4.67 (0.22,97.03)	4.65 (0.24,89.57)	5.35 (0.26,108.81)	5.94 (0.28,124.22)	6.82 (0.37,126.19)	7.00 (0.39,126.64)	7.19 (0.35,146.17)	9.11 (0.45,182.94)	11.17 (0.54,229.01)
0.25 (0.01,5.26)	VOCZ + ANFG	1.17 (0.65,2.11)	1.16 (0.38,3.56)	1.34 (0.86,2.09)	1.49 (0.81,2.74)	1.71 (0.62,4.68)	1.75 (0.69,4.48)	1.80 (0.90,3.62)	2.28 (1.21,4.30)	2.80 (0.90,8.69)
0.21 (0.01,4.45)	0.85 (0.47,1.54)	ISCZ	1.00 (0.33,2.96)	1.15 (0.78,1.69)	1.27 (0.71,2.26)	1.46 (0.55,3.87)	1.50 (0.61,3.70)	1.54 (0.80,2.94)	1.95 (1.09,3.49)	2.39 (0.79,7.22)
0.22 (0.01,4.14)	0.86 (0.28,2.63)	1.00 (0.34,2.99)	VOCZ + CAFG	1.15 (0.41,3.22)	1.28 (0.42,3.88)	1.47 (0.72,2.98)	1.51 (0.82,2.76)	1.55 (0.55,4.32)	1.96 (0.73,5.28)	2.40 (0.84,6.88)
0.19 (0.01,3.80)	0.75 (0.48,1.16)	0.87 (0.59,1.29)	0.87 (0.31,2.42)	VOCZ	1.11 (0.73,1.69)	1.27 (0.51,3.15)	1.31 (0.57,2.99)	1.34 (0.78,2.30)	1.70 (1.08,2.68)	2.09 (0.73,5.92)
0.17 (0.01,3.53)	0.67 (0.36,1.24)	0.79 (0.44,1.40)	0.78 (0.26,2.38)	0.90 (0.59,1.38)	POCZ	1.15 (0.42,3.12)	1.18 (0.47,2.99)	1.21 (0.61,2.41)	1.54 (0.83,2.86)	1.88 (0.61,5.80)
0.15 (0.01,2.72)	0.59 (0.21,1.61)	0.69 (0.26,1.82)	0.68 (0.34,1.39)	0.79 (0.32,1.94)	0.87 (0.32,2.37)	ABLC	1.03 (0.71,1.48)	1.05 (0.43,2.61)	1.34 (0.56,3.17)	1.64 (0.64,4.18)
0.14 (0.01,2.58)	0.57 (0.22,1.46)	0.67 (0.27,1.65)	0.66 (0.36,1.22)	0.76 (0.33,1.75)	0.85 (0.33,2.15)	0.97 (0.67,1.41)	L-AmB	1.03 (0.45,2.36)	1.30 (0.59,2.85)	1.60 (0.68,3.77)
0.14 (0.01,2.83)	0.56 (0.28,1.12)	0.65 (0.34,1.24)	0.65 (0.23,1.81)	0.74 (0.43,1.28)	0.83 (0.42,1.64)	0.95 (0.38,2.35)	0.97 (0.42,2.23)	ABCD	1.27 (0.96,1.67)	1.55 (0.53,4.54)
0.11 (0.01,2.20)	0.44 (0.23,0.83)	0.51 (0.29,0.92)	0.51 (0.19,1.37)	0.59 (0.37,0.93)	0.65 (0.35,1.21)	0.75 (0.31,1.78)	0.77 (0.35,1.68)	0.79 (0.60,1.04)	AmB	1.23 (0.43,3.46)
0.09 (0.00,1.84)	0.36 (0.12,1.11)	0.42 (0.14,1.26)	0.42 (0.15,1.19)	0.48 (0.17,1.36)	0.53 (0.17,1.64)	0.61 (0.24,1.55)	0.63 (0.27,1.48)	0.64 (0.22,1.88)	0.82 (0.29,2.30)	CAFG

Abbreviations: AmB, amphotericin B deoxycholate; L-AmB, liposomal amphotericin B; ABCD, amphotericin B colloidal dispersion; ABLC, amphotericin B lipid complex; VOCZ, voriconazole; POCZ, posaconazole; CAFG, caspofungin; ISCZ, isavuconazole; ANFG, anidulafungin

Regarding mortality rate, the approach that ranked highest with the lower mortality rate was liposomal amphotericin B plus caspofungin (SUCRA, 88.2%; mean rank, 2.2), followed by voriconazole plus anidulafungin (SUCRA, 79.1%; mean rank, 3.1), isavuconazole (SUCRA, 69.1%; mean rank, 4.1), voriconazole plus caspofungin (SUCRA, 66.9%; mean rank, 4.3), voriconazole (SUCRA, 57.7%; mean rank, 5.2), posaconazole (SUCRA, 48.4%; mean rank, 6.2). Caspofungin ranked the lowest (SUCRA, 12.9%; mean rank, 9.7). Compared to voriconazole, liposomal amphotericin B plus caspofungin also showed a trend in reducing mortality (RR, 0.19; 95% CI, 0.01–3.80), but the difference is not significant. Voriconazole plus anidulafungin (RR, 0.75; 95% CI, 0.48–1.16), voriconazole plus caspofungin (RR, 0.87; 95% CI, 0.31–2.42), isavuconazole (RR, 0.87; 95% CI, 0.59–1.29), posaconazole (RR, 1.11; 95% CI, 0.73–1.69) didn’t show a significant difference compared with voriconazole (Table 3).

### Funnel plot and publication bias

**Fig. 3:**
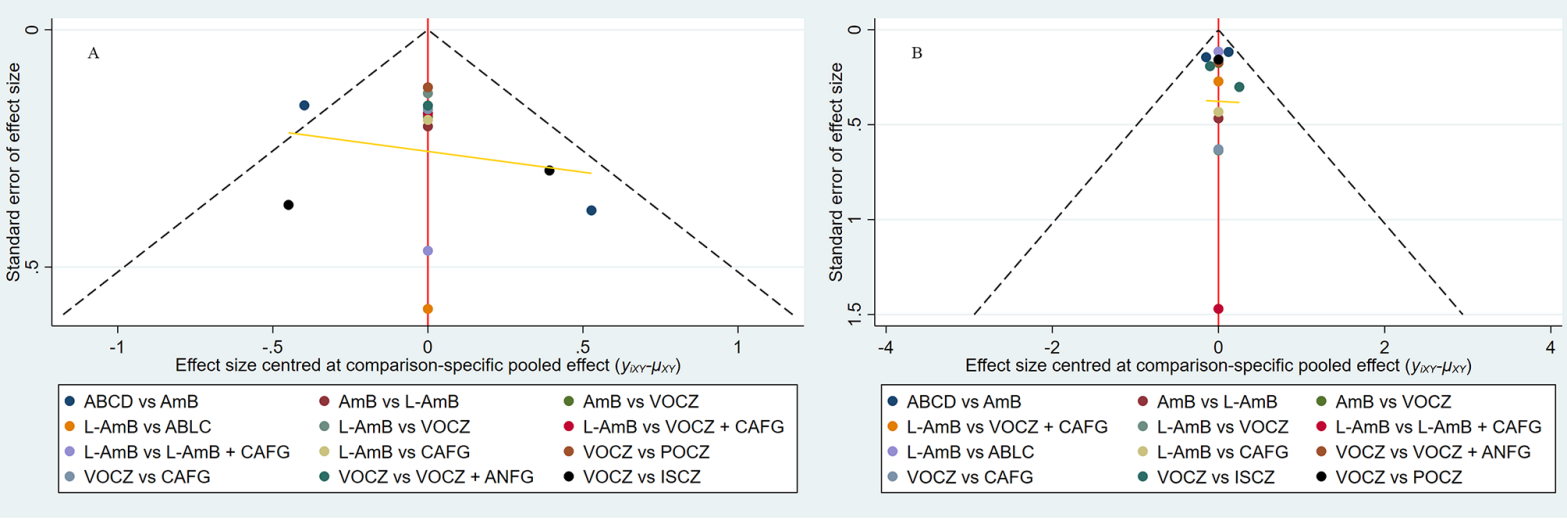
Funnel Plots of all outcomes. Funnel Plots of response (**A**) and mortality (**B**). In the comparison-adjusted funnel plot, the horizontal axis shows the difference of each i-study estimate YiXY from the summary effect for the respective comparison (YiXY-µXY) while the vertical axis presents the measure of dispersion of YiXY, namely the standard error of the effect size. The red line shows the null hypothesis. Each point represents a direct comparison; different colors correspond to different comparisons. The dashed black line represents the 95% confidence interval. The horizontal line represents the regression line; the regression line demonstrates that no asymmetry is present. AmB, amphotericin B deoxycholate; L-AmB, liposomal amphotericin B; ABCD, amphotericin B colloidal dispersion; ABLC, amphotericin B lipid complex; VOCZ, voriconazole; POCZ, posaconazole; CAFG, caspofungin; ISCZ, isavuconazole; ANFG, anidulafungin

Heterogeneity and inconsistency are shown in Table 4. Heterogeneity was low for mortality; heterogeneity was reasonable for the response. Loop inconsistency for liposomal amphotericin B, voriconazole, and caspofungin was not found for response and mortality (eFigure 3). As shown in Fig. 3, it did not suggest any publication bias in the comparison-adjusted funnel plots.

**Table 4:** Tests for Inconsistency, Heterogeneity, and Small-Study Effects

Outcome	Inconsistency at the overall level		Heterogeneity (τ2)	Egger’s test *P* value
	χ2	*P* value		
Response	0.08	0.78	0.15	0.38
Mortality	2.21	0.14	0.02	0.27

## Discussion

In the present NMA, we combined direct and indirect evidence to compare primary therapies for patients with IA. Our result may provide vital information for these patients’ clinical decision-making for antifungal therapy. We derived some principal findings from our analysis: combination antifungal agents of liposomal amphotericin B and caspofungin may be the best choice for primary therapy of IA, and voriconazole plus echinocandins also could be considered. Isavuconazole and voriconazole are the top choices for primary therapy, intravenous or oral posaconazole can be considered an alternative agent. Isavuconazole and voriconazole are recommended for IA as primary treatment for solid-organ and hematopoietic stem cell transplant patients according to the international guidelines [8, 9]. Overall, isavuconazole, voriconazole, posaconazole, liposomal amphotericin B plus caspofungin and voriconazole plus echinocandins are recommended as the most reasonable options for the primary therapy of IA.

In the current NMA, liposomal amphotericin B plus caspofungin demonstrated the highest response rate and lowest mortality rate. However, it should be noted that the evidence supporting this finding is based on a pilot study with a limited sample size [14]. The study showed that the combination therapy group exhibited significantly more favourable responses (partial or complete) than the liposomal amphotericin B monotherapy group (10/15 vs. 4/15). Moreover, at week 12, survival rate was higher in the combination therapy group (100%) than in the amphotericin B monotherapy group (80%). A larger randomized controlled trial is necessary to confirm these findings. Therefore, liposomal amphotericin B plus caspofungin is not recommended as a primary therapy according to international guidelines [8, 9]. 

Our NMA concluded that combination antifungal therapy of voriconazole and an echinocandin don’t show more efficacy than monotherapy with voriconazole, isavuconazole, or posaconazole. A large RCT had assessed the safety and efficacy of 6 weeks of voriconazole with or without anidulafungin for the treatment of IA in patients with hematologic malignancies and/or hematopoietic cell transplantation, mortality rates at 6 weeks were 19.3% (26/135) for combination therapy and 27.5% (39/142) for monotherapy (difference, -8.2%, 95% CI, -19.0 to 1.5; *P* = 0.087) [13]. Another observational study compared the primary therapy of 40 solid organ transplant patients who received voriconazole and caspofungin with a historical control group of 47 patients who were given liposomal amphotericin B. Overall survival at 90 days was 67.5% (27/40) in the cases and 51% (24/47) in the control group (HR 0.57, 95% CI: 0.29–1.1, *P* = 0.11) [15]. Overall, due to limited evidence of the effectiveness of combination therapy, it is essential for clinicians to carefully evaluate the potential risks and benefits of administering two drugs. This evaluation should consider individual factors such as potential toxicities, the severity of the disease, and the level of immunosuppression, as well as practical considerations, including cost and the feasibility of intravenous therapy.

Based on the Global Comparative Aspergillus Study results [16, 32], primary therapy with voriconazole showed better responses and improved survival and resulted in fewer severe side effects than the standard approach with amphotericin B in patients with IA. Voriconazole is the preferred antifungal agent recommended by international guidelines [8, 9]. Our NMA found that isavuconazole and posaconazole are equally effective in treating IA as voriconazole. However, while isavuconazole is recommended as the preferred antifungal agent by international guidelines, posaconazole is not. Because published in 2016, the SECURE trial showed isavuconazole was non-inferior to voriconazole for the primary therapy of suspected invasive mold disease [33]. Before the latest vision guidelines were updated, no RCT had compared the efficacy of posaconazole and voriconazole. Now, posaconazole injection and delayed-release tablets are FDA approved for the treatment of IA in patients ≥ 13 years because a RCT comparing posaconazole vs. voriconazole for the therapy of IA in 575 patients, all-cause mortality at day 42 was lower in the posaconazole group (15% vs. 21%, *p* < 0.0001) in the intention to treat analysis [10]. For primary therapy of IA, we recommend voriconazole, isavuconazole, and posaconazole, if resistant Aspergillus isolates are not suspected. When patients cannot tolerate voriconazole and avoid its toxicity, Posaconazole and isavuconazole are the preferred alternatives [35]. It is noted that the absorption of an oral suspension of posaconazole is less reliable than the delayed-release tablets, and patients with higher serum posaconazole levels have higher favourable response rates than those with lower drug levels [36]. We don’t recommend oral suspension of posaconazole for primary therapy in patients with IA if posaconazole injection and delayed-release tablets are available.

Our NMA included 4 formulations of amphotericin B, liposomal amphotericin B, amphotericin B colloidal dispersion, and amphotericin B lipid complex were ranked higher than amphotericin B deoxycholate at response and mortality in our analysis. And amphotericin B deoxycholate was ranked lowest at response and near lowest at mortality among all the 11 antifungal agents. Amphotericin B deoxycholate should not be considered in the primary therapy of IA. The other 3 formulations of amphotericin B have similar efficacy, but they all have less effect than voriconazole, posaconazole, and isavuconazole. The echinocandins, including caspofungin and anidulafungin, are encompassed within our analysis. Though the FDA has approved caspofungin for treating IA in patients who cannot tolerate or are refractory to standard therapy [37], monotherapy with caspofungin was ranked lowest at mortality in the rank analysis.

Though we couldn’t analyze antifungal agent toxicity due to limited data, clinical practice demands considering drug toxicity alongside efficacy. Amphotericin B deoxycholate often triggers severe nephrotoxicity due to vasoconstrictive and tubulo-toxic effects [27–30, 38–40]. Lipid formulations mitigate, but don’t eliminate, nephrotoxicity [41, 42]. Two hypotheses suggest: absence of deoxycholate in liposomal preparations reduces tubular toxicity, and liposomes may preferentially target fungal sites, sparing kidney cells [38, 40]. Concurrent administration of nephrotoxic agents, baseline chronic kidney disease, and dose-dependent factors heighten nephrotoxicity risk for both formulations [43–45]. Additionally, amphotericin B can also disrupt electrolyte and acid-base balance, necessitating electrolyte supplement for management [38]. Triazoles pose lower nephrotoxicity concerns than amphotericin B, with commonly reported gastrointestinal and hepatic function abnormalities [10, 16, 33]. Each azole carries unique toxicity profiles; Voriconazole is linked with vision changes, neurologic toxicity, skin toxicity, periostitis, cardiac toxicity, alopecia, and nail changes. Isavuconazole shares similar toxicity with voriconazole but at a lower rate. Posaconazole has a more favorable toxicity profile, particularly regarding cardiac toxicity and vision changes [10, 33, 46]. . Echinocandins are generally well tolerated, with less frequent serious toxicity than other systemic antifungal agents [46]. Additionally, route of administration is crucial; with the minimum 12-week therapy duration, both amphotericin B and echinocandins are exclusively administered intravenously, potentially leading to prolonged hospital stays and increased costs.

Finally, while our NMA revealed triazoles’ favorable efficacy for antifungal therapy, the mortality rate of invasive aspergillosis persists at up to 20% [10, 33]. Despite the severity of invasive fungal infections, research funding in this field has been perennially lacking, leading to inadequate diagnostic and therapeutic options. However, hope is on the horizon with the emergence of new antifungal agents, such as rezafungin, ibrexafungerp, olorofim, and fosmanogepix, which are advancing to advanced trial stages or gaining approval from regulatory bodies, following a prolonged period of near emptiness in the drug pipeline [47, 48]. 

### Limitations

Our study has some limitations. The first limitation is the lack of enough head-to-head RCTs in IA. To overcome this limitation, we included non-randomized trials in our NMA. Due to constraints in available data, we employed a naive data synthesis method instead of the more robust statistical methodologies recommended by the guideline [49]. The insufficiencies in the study design and inconsistent conduct and reporting of the included studies have significantly influenced the outcomes of our analysis. Second, the different durations of treatment and follow-up times are also significant factors contributing to heterogeneity. Third, the definitions for the response have some different criteria among the included studies in our NAM, which could influence our statistical result. Fourth, more than 60% of the studies included less than 100 patients per group, which could lead to bias due to small study effects. Fifth, the characteristics of patients and treatments were heterogeneous among the various RCTs and observational studies. Due to insufficient data, we could not perform more detailed subgroup analyses, such as those for different underlying diseases. We also could not conduct a more detailed analysis considering the dosage form and dose of agents. As a result, the evidence gathered from this NMA should be approached with caution when making shared decisions. Nonetheless, our research offers valuable insights that could help shape future practice-changing prospective trials.

## Conclusions

This network meta-analysis assessed the efficacy of various antifungal agents as primary treatment in patients with IA. Our findings suggest that voriconazole, posaconazole, and isavuconazole may be the best options for patients with IA, liposomal amphotericin B plus caspofungin may also be a reasonable option.

### Electronic supplementary material

Below is the link to the electronic supplementary material.

Supplementary Material 1

## Data Availability

The data of this study can be obtained from the corresponding author according to reasonable requirements.
