# Supplementary material for: Compare the efficacy of antifungal agents as primary therapy for invasive aspergillosis: a network meta-analysis
Source: BMC Infect Dis. 2024 Jun 12;24:581. doi: 10.1186/s12879-024-09477-9 (PMC11170913; doi:10.1186/s12879-024-09477-9)
Supplement: Supplementary file 1 — Supplementary Material 1 [file 12879_2024_9477_MOESM1_ESM.docx]

Supplemental file of compare the efficacy of antifungal agents as primary therapy for invasive aspergillosis: a network meta-analysis

eTable 1. Literature search strategy

**eTable 2**: Newcastle Ottawa score to assess the quality of the included studies

**eFigure 1**. Revised tool for risk of bias to assess the quality of the included randomized clinical trial

**eFigure 2**. Forest Plots of all outcomes.

eFigure 3. Inconsistency Plot of All Outcomes

eTable 1. Literature search strategy

| Search results of Pubmed | | |
| --- | --- | --- |
| #1 | "invasive"[Title/Abstract] | 457615 |
| #2 | "Aspergillosis"[MeSH Terms] OR "Aspergillus"[MeSH Terms] OR "aspergill*"[Title/Abstract] | 69282 |
| #3 | "Antifungal Agents"[MeSH Terms] OR "Antifungal"[Title/Abstract] OR "Amphotericin B"[MeSH Terms] OR "Amphotericin B"[Title/Abstract] OR "Itraconazole"[MeSH Terms] OR "Itraconazole"[Title/Abstract] OR "Voriconazole"[MeSH Terms] OR "Voriconazole"[Title/Abstract] OR "posaconazole"[Title/Abstract] OR "isavuconazole"[Title/Abstract] OR "Anidulafungin"[MeSH Terms] OR "Anidulafungin"[Title/Abstract] OR "Caspofungin"[MeSH Terms] OR "Caspofungin"[Title/Abstract] OR "Micafungin"[MeSH Terms] OR "Micafungin"[Title/Abstract] | 113279 |
| #4 | "response"[Title/Abstract] OR "mortality"[Title/Abstract] OR "survival"[Title/Abstract] | 4224878 |
| #5 | #1 AND #2 AND #3 AND #4 | 2555 |
| Search results of Emabse | | |
| #1 | invasive:ab,ti | 680160 |
| #2 | 'aspergillosis'/exp OR 'aspergillus'/exp OR 'aspergill*':ab,ti | 106878 |
| #3 | 'antifungal agent'/exp OR 'antifungal':ab,ti OR 'amphotericin b'/exp OR 'amphotericin b':ab,ti OR 'itraconazole'/exp OR 'itraconazole':ab,ti OR 'voriconazole'/exp OR 'voriconazole':ab,ti OR 'posaconazole'/exp OR 'posaconazole':ab,ti OR 'isavuconazole'/exp OR 'isavuconazole':ab,ti OR 'anidulafungin'/exp OR 'anidulafungin':ab,ti OR 'caspofungin'/exp OR 'caspofungin':ab,ti OR 'micafungin'/exp OR 'micafungin':ab,ti | 471761 |
| #4 | 'response':ab,ti OR 'mortality':ab,ti OR 'survival':ab,ti | 470146 |
| #5 | 'crossover procedure':de OR 'double-blind procedure':de OR 'randomized controlled trial':de OR 'single-blind procedure':de OR random*:de,ab,ti OR factorial*:de,ab,ti OR crossover*:de,ab,ti OR ((cross NEXT/1 over*):de,ab,ti) OR placebo*:de,ab,ti OR ((doubl* NEAR/1 blind*):de,ab,ti) OR ((singl* NEAR/1 blind*):de,ab,ti) OR assign*:de,ab,ti OR allocat*:de,ab,ti OR volunteer*:de,ab,ti | 5724993 |
| #6 | #1 AND #2 AND #3 AND #4 | 4680 |
| Search results of CENTRAL | | |
| #1 | (invasive):ti,ab,kw | 39501 |
| #2 | MeSH descriptor: [Aspergillosis] explode all trees | 249 |
| #3 | MeSH descriptor: [Aspergillus] explode all trees | 122 |
| #4 | (aspergill*):ti,ab,kw | 963 |
| #5 | #1 AND (#2 OR #3 OR #4) | 347 |
| #6 | MeSH descriptor: [Antifungal Agents] explode all trees | 2038 |
| #7 | (Antifungal):ti,ab,kw | 3638 |
| #8 | MeSH descriptor: [Amphotericin B] explode all trees | 616 |
| #9 | ("Amphotericin B"):ti,ab,kw | 1232 |
| #10 | MeSH descriptor: [Itraconazole] explode all trees | 598 |
| #11 | (Itraconazole):ti,ab,kw | 1181 |
| #12 | MeSH descriptor: [Voriconazole] explode all trees | 238 |
| #13 | (Voriconazole):ti,ab,kw | 491 |
| #14 | (posaconazole):ti,ab,kw | 189 |
| #15 | (isavuconazole):ti,ab,kw | 66 |
| #16 | MeSH descriptor: [Anidulafungin] explode all trees | 41 |
| #17 | (Anidulafungin):ti,ab,kw | 81 |
| #18 | MeSH descriptor: [Caspofungin] explode all trees | 89 |
| #19 | (Caspofungin):ti,ab,kw | 194 |
| #20 | MeSH descriptor: [Micafungin] explode all trees | 81 |
| #21 | (Micafungin):ti,ab,kw | 164 |
| #22 | #5 OR #7 OR #8 OR #9 OR #10 OR #11 OR #12 OR #13 OR #14 OR #15 OR #16 OR #17 OR #18 OR #19 OR #20 OR #21 | 5219 |
| #23 | #5 AND #22 | 293 |

**eTable 2**: Newcastle Ottawa score to assess the quality of the included studies

| **Reference** | **Selection** | **Comparability** | **Outcome** | **Overall** |
| --- | --- | --- | --- | --- |
| White, et al 1997 | 4 | 0 | 3 | 7 |
| Leenders, et al 1998 | 4 | 2 | 3 | 9 |
| Singh, et al 2006 | 4 | 2 | 3 | 9 |
| Caillot, et al 2007 | 4 | 2 | 3 | 9 |
| Hachem, et al 2008 | 3 | 2 | 3 | 8 |
| Pagano, et al 2010 | 4 | 2 | 3 | 9 |
| Cheng, et al 2020 | 3 | 2 | 3 | 8 |

**eFigure 1**. Revised tool for risk of bias to assess the quality of the included randomized clinical trial


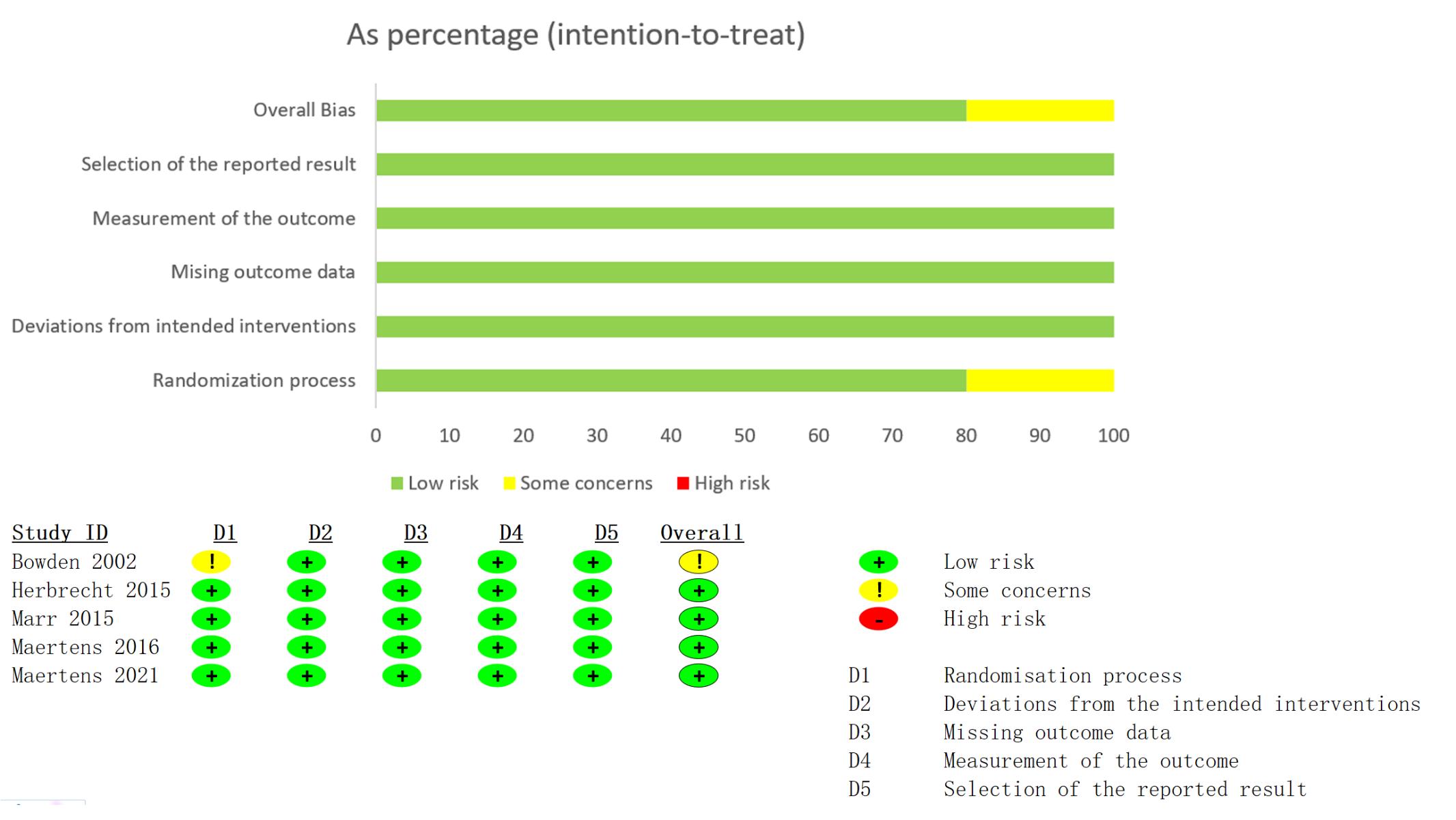


**
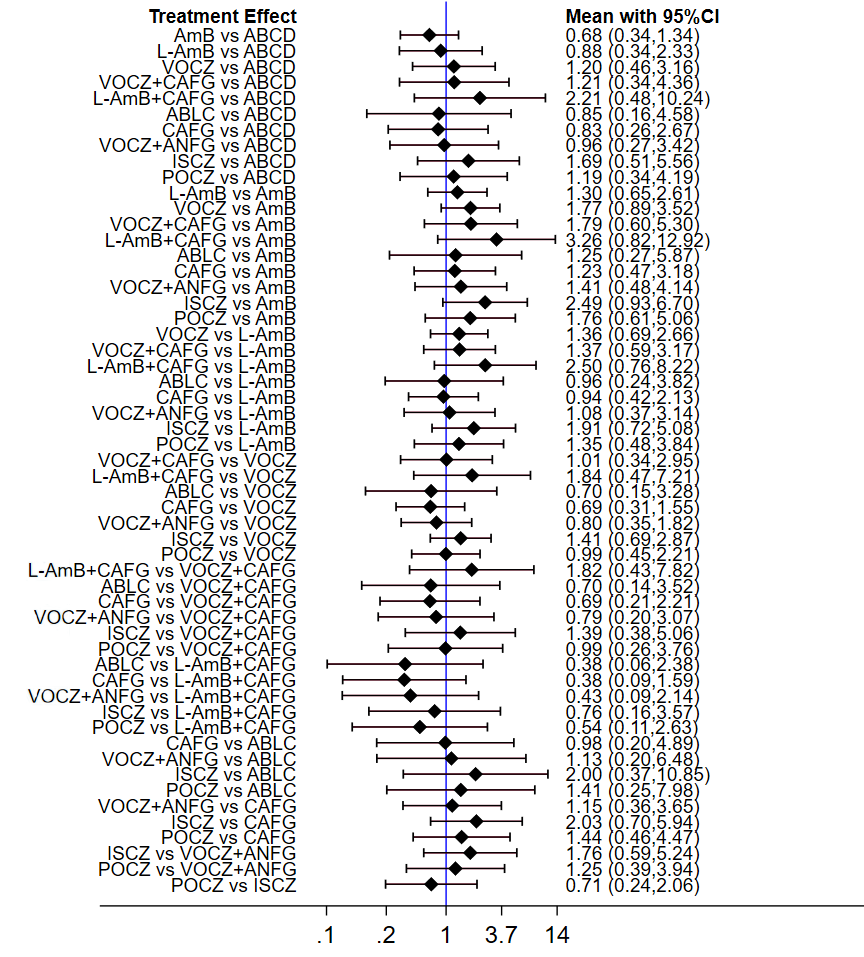
eFigure 2**. Forest Plots of all outcomes.

**Panel A.** Response

The results are presented in the form of Relative risks (RRs) and 95% confidence intervals (CIs). The blue line, which represents the null effect, has a value of 1. The solid black lines indicate the CIs, while the diamond symbolizes the RRs. To interpret the forest plot for each pairwise comparison, start with the diamond. If the diamond and the entire CI do not reach the blue line of null effect, it means there is a significant difference. If the entire CI lies on the left side of the null effect, the event occurs more frequently in the "intervention arm". Conversely, if the entire CI is on the right side, the event is statistically more frequent in the "reference arm." If the entire CI crosses the line of null effect, then there is no significant difference between the two procedures. AmB, amphotericin B deoxycholate; L-AmB, liposomal amphotericin B; ABCD, amphotericin B colloidal dispersion; ABLC, amphotericin B lipid complex; VOCZ, voriconazole; POCZ, posaconazole; CAFG, caspofungin; ISCZ, isavuconazole; ANFG, anidulafungin.


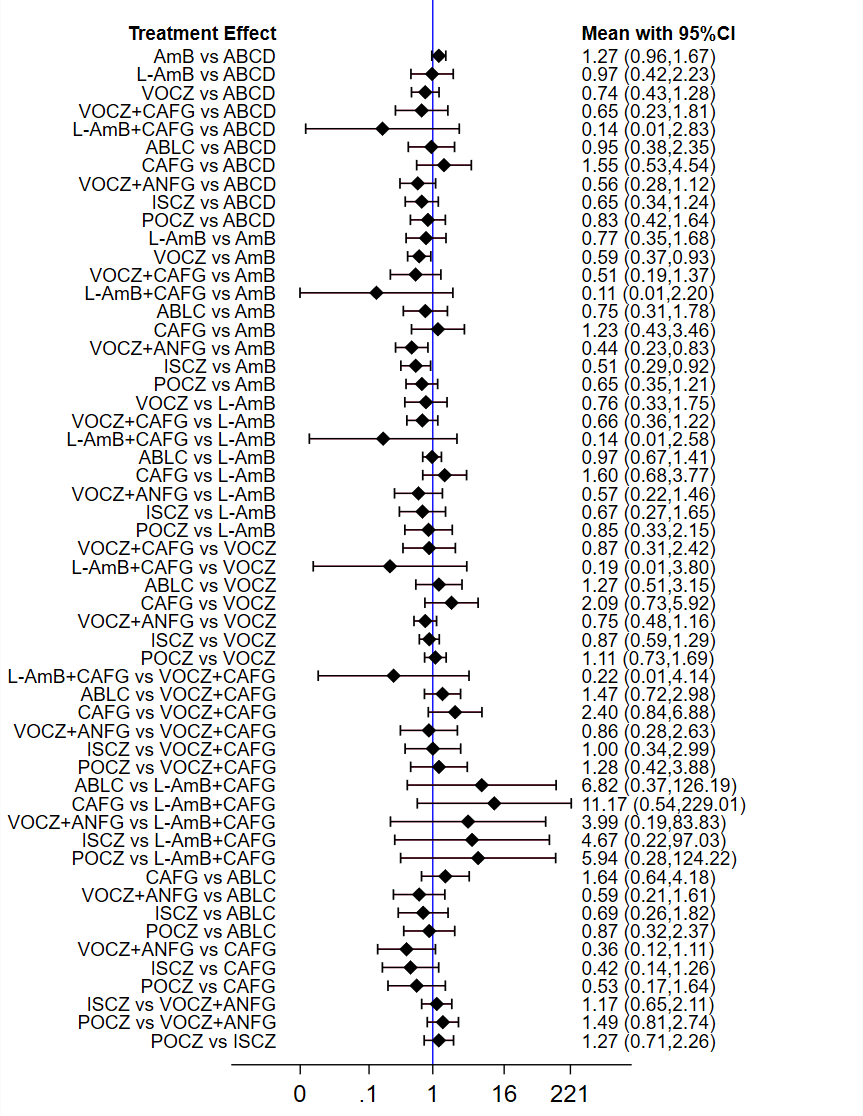
**eFigure 2**. Forest Plots of all outcomes.

**Panel B.** mortality

The results are presented in the form of Relative risks (RRs) and 95% confidence intervals (CIs). The blue line, which represents the null effect, has a value of 1. The solid black lines indicate the CIs, while the diamond symbolizes the RRs. To interpret the forest plot for each pairwise comparison, start with the diamond. If the diamond and the entire CI do not reach the blue line of null effect, it means there is a significant difference. If the entire CI lies on the left side of the null effect, the event occurs more frequently in the "intervention arm". Conversely, if the entire CI is on the right side, the event is statistically more frequent in the "reference arm." If the entire CI crosses the line of null effect, then there is no significant difference between the two procedures. AmB, amphotericin B deoxycholate; L-AmB, liposomal amphotericin B; ABCD, amphotericin B colloidal dispersion; ABLC, amphotericin B lipid complex; VOCZ, voriconazole; POCZ, posaconazole; CAFG, caspofungin; ISCZ, isavuconazole; ANFG, anidulafungin.

eFigure 3. Inconsistency Plot of All Outcomes


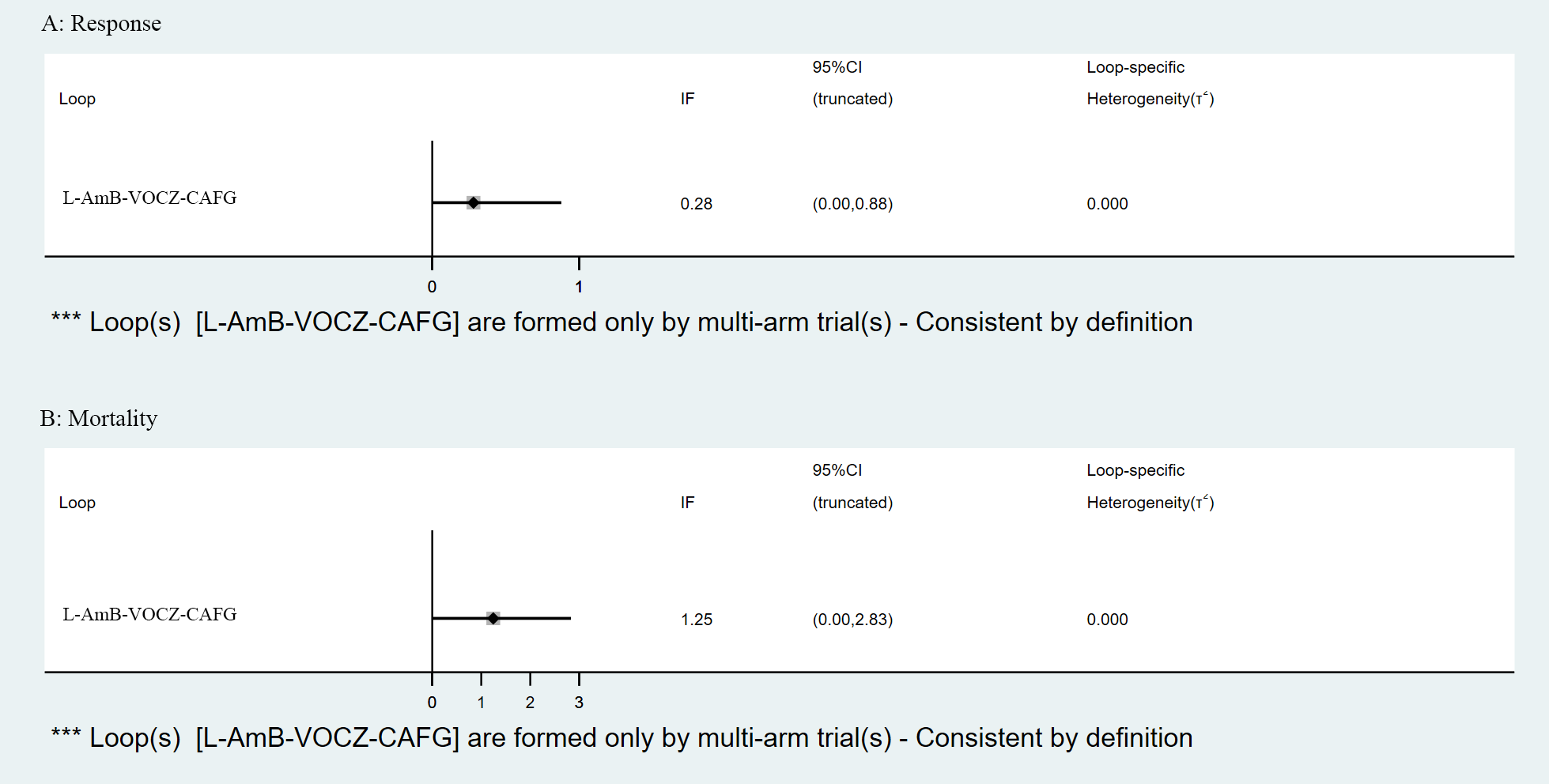


Inconsistency plot assuming loop-specific heterogeneity estimates using the method of moments estimator. L-AmB, liposomal amphotericin B; VOCZ, voriconazole; CAFG, caspofungin.
